# Evaluating the Causal Role of Genetically Inferred Immune Cells and Inflammatory Cytokines on Myalgic Encephalomyelitis/Chronic Fatigue Syndrome

**DOI:** 10.3390/biomedicines13051200

**Published:** 2025-05-15

**Authors:** Lincheng Duan, Jingyi Yang, Junxin Zhao, Zhuoyang Chen, Hong Yang, Dingjun Cai

**Affiliations:** 1 Acupuncture and Tuina School, Chengdu University of Traditional Chinese Medicine, Chengdu 611137, China; lincheng@stu.cdutcm.edu.cn (L.D.); yangjingyi@stu.cdutcm.edu.cn (J.Y.); xiaoyuzhao631@gmail.com (J.Z.); chenzhuoyang2025@163.com (Z.C.); yanghong95ob@163.com (H.Y.); 2 Key Laboratory of Acupuncture for Senile Disease, Chengdu University of TCM, Ministry of Education, Acupuncture and Chronobiology Key Laboratory of Sichuan Province, Chengdu 611137, China

**Keywords:** inflammatory cytokines, ME/CFS, immune cell, mendelian randomization, immune system dysfunction

## Abstract

**Background/Objectives**: Myalgic encephalomyelitis/chronic fatigue syndrome (ME/CFS) is a multifaceted and diverse disorder with an ambiguous etiology. Recent evidence indicates that immune system impairment and inflammatory mechanisms are pivotal to the initiation and advancement of ME/CFS. Nonetheless, the causal relationships among these factors remain inadequately comprehended. **Methods**: This study investigated the causative contributions of immunological dysfunction and inflammatory variables in ME/CFS utilizing genome-wide association study (GWAS) data. We employed Mendelian randomization (MR) to investigate associations between 91 inflammatory cytokines, 731 immune cell characteristics, and the risk of ME/CFS. Summary statistics for immune cell traits and inflammatory cytokines were sourced from European GWAS cohorts (n = 3757 and n = 14,824, respectively), while ME/CFS data were obtained from the UK Biobank (n = 462,933, including 2076 cases). We predominantly employed the inverse variance weighted (IVW) approach, complemented by MR-Egger, weighted median, BWMR, and MR-RAPS tests to guarantee robust and precise outcomes. **Results**: The study revealed significant causal links between various inflammatory factors, immune cell characteristics, and the risk of ME/CFS. Increased CXCL5 and CCL20 levels were significantly linked to a higher risk of ME/CFS, while elevated TNF levels were inversely related to ME/CFS risk. Furthermore, 13 immune cell characteristics were identified as having substantial causal associations with the likelihood of ME/CFS. These data are supportive of the causality that immune system dysfunction and inflammatory variables play a pivotal role in the development of ME/CFS. **Conclusions**: This study provides new insights into the causal role of immune system dysfunction in the development of ME/CFS, contributing to a deeper understanding of its underlying mechanisms. These results offer a foundation for identifying diagnostic biomarkers and developing targeted therapeutic strategies. Future research should validate these findings using multi-center cohort studies and further investigate the mechanisms behind key factors to enable the development of personalized treatment approaches.

## 1. Introduction

The complex, long-term illness known as myalgic encephalomyelitis/chronic fatigue syndrome (ME/CFS) is typified by post-exertional malaise (PEM), myalgia (muscle pain), cognitive impairments, and a variety of other symptoms like fever, pain, irritable bowel syndrome, and immune system abnormalities [1]. All of these symptoms adversely affect patients’ quality of life [2]. Before the COVID-19 pandemic, ME/CFS impacted around 1 to 2.5 million individuals in the US, with patients indicating a diminished quality of life relative to those suffering from conditions like multiple sclerosis or chronic kidney disease [3,4]. Following the pandemic, ME/CFS incidence has risen, and its clinical presentation has been noted to closely resemble that of post-COVID syndrome [5,6]. Despite the substantial impact of ME/CFS on patients’ lives, its underlying etiology and pathological mechanisms remain poorly understood.

Emerging evidence increasingly highlights a strong association between ME/CFS and immune system dysfunction. For instance, single-cell RNA sequencing studies of PEM in ME/CFS patients have identified significant dysregulation of monocyte function, with the number of dysregulated monocytes positively correlated with disease severity [7]. Immunological exhaustion, senescence, and hypersensitivity in ME/CFS patients have been linked to impaired functionality of immune cells, particularly T cells [8]. Other findings include increased T cell apoptosis, elevated levels of necrotic cell death, and structural and functional abnormalities in organelles [9]. While these studies emphasize the role of immune cells in ME/CFS pathophysiology, their findings often lack consistency, potentially due to differences in study design, individual patient heterogeneity, or methodological variations. This inconsistency underscores a predominant focus on correlational rather than causal relationships in current research. Additionally, the absence of reliable diagnostic biomarkers and effective therapeutic strategies for ME/CFS contributes to misdiagnosis and underrecognition of the disease [10]. Establishing a clear causal link between immune cell dysfunction and ME/CFS is critical for advancing our understanding of the disease’s underlying causes, identifying potential biomarkers, and discovering novel treatment targets.

This study applied Mendelian randomization (MR) to investigate the causal relationships involving immune cell function, inflammatory factors, and ME/CFS, aiming to bridge current gaps in scientific understanding. MR is an analytical method grounded in Mendel’s principles of heredity, employing genetic variants as instrumental variables (IVs) to ascertain if the observed correlations between exposures and outcomes are causative [11]. This approach minimizes confounding factors and reverse causation bias, as genetic variants are not influenced by environmental, social, or behavioral factors [12,13]. Based on the fact that studies have shown abnormalities in the immune system of patients with ME/CFS, including changes in TNF levels and B-cell dysfunction, we hypothesized that dysregulation of the TNF pathway and B-cell-associated immune pathway would be causally related to the development of ME/CFS. This analysis seeks to evaluate whether immune cell dysfunction and inflammatory factors are key contributors to the development of ME/CFS, examine the potential mediating role of inflammatory markers in these processes, and offer meaningful insights for the discovery of diagnostic biomarkers and the advancement of targeted treatment approaches.

## 2. Materials and Methods

### 2.1. Study Design

**Figure 1:**
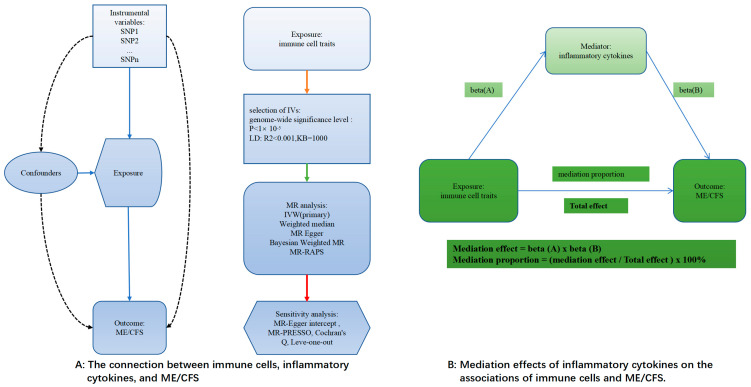
An illustration of the principles and procedure of the analysis. The solid line indicates a clear effect downstream through this line, the dashed line indicates no effect, the color of the line has no special meaning, and β(A/B) indicates the effect value.

The two-sample MR method was used in this study to look into the links between ME/CFS, 91 circulating inflammatory cytokines, and 731 immune cell characteristics (Figure 1A). A two-step MR study was conducted to investigate if circulating inflammatory cytokines act as mediators in the relationship between immune cell characteristics and ME/CFS (Figure 1B).

### 2.2. Data Sources

#### 2.2.1. Datasets for Circulating Inflammatory Cytokines and Immune Cell

Summary statistics for immune cell traits were sourced from Orrù V et al. [14]. Data from 3757 European people served as the basis for the first GWAS on immunological characteristics, derived from flow cytometry and transcriptomic profiling of 731 immune phenotypes in European individuals. Information on 91 circulating inflammatory cytokines was obtained through a meta-analysis measured via multiplex immunoassays conducted on 14,824 individuals of European descent [15].

#### 2.2.2. Datasets for ME/CFS

Additionally, we incorporated data from the UK Biobank, specifically from a study on non-cancer diseases (self-reported: chronic fatigue syndrome), selecting data related to ME/CFS, which included 462,933 individuals (case = 2076, control = 460,857). ME/CFS was a self-reported diagnosis from the UK Biobank, with cases confirmed using CDC criteria. Further details are provided in Table 1. Although the diagnosis of ME/CFS in this study was based on patient self-report, previous studies have validated the validity of self-reported data in studies of similar diseases [16,17].

### 2.3. IV Selection

To identify appropriate instrumental variables (IVs) for MR analysis, single nucleotide polymorphisms (SNPs) were selected based on their meeting the genome-wide significance threshold (*p* < 1 × 10^−5^) [18,19]. SNPs located within a 10,000 kb genomic region and exhibiting an r^2^ value greater than 0.001 were excluded to minimize the effects of linkage disequilibrium. The association strength between IVs and exposure factors was evaluated using the F-statistic. SNPs with F-values below 10 were classified as weak instruments and excluded from the analysis. F-statistics were calculated following established formulas in previous studies [20].

### 2.4. MR Analysis

The inverse variance weighted (IVW) method served as the primary analytical approach in this study to explore the causal associations between immune cells, inflammatory factors, and ME/CFS [21]. The IVW method is widely used in MR analysis and provides an overall estimate by combining data from multiple genetic variants through a weighted approach. To strengthen the robustness and reliability of the findings, additional analytical techniques were employed. The weighted median (WM) method was utilized to generate reliable causal effect estimates, assuming that at least 50% of the total weight was derived from valid instrumental variables [22]. Additionally, MR-Egger regression was applied to assess the presence of directional pleiotropy and to produce causal estimates adjusted for potential bias [23]. The relevance of pleiotropy was assessed using the *p*-value of the intercept term in MR-Egger regression; *p* > 0.05 denoted negligible pleiotropic effects. Cochran’s Q test was conducted to evaluate heterogeneity among IVs, which helped evaluate potential variability in SNP distributions. The reliability of the causal estimations was increased by using the MR-PRESSO approach to further detect horizontal pleiotropy and eliminate possible outliers [24].

To address potential biases from weak instruments and pleiotropy, the study utilized MR-RAPS [25] and Bayesian weighted MR (BWMR) [26] methods. MR-RAPS offers reliable causal effect estimates despite weak instruments and horizontal pleiotropy, whereas BWMR enhances causal inference by addressing pleiotropy-induced violations of instrumental variable assumptions, particularly in weak-effect contexts. The leave-one-out (LOO) method was utilized for sensitivity analyses to evaluate the impact of individual SNPs on the overall causal estimates. The Steiger directionality test was also conducted to evaluate the potential impact of reverse causation [27]. Any SNPs identified as having reverse causal effects were manually excluded from the analysis to ensure the validity of the findings.

### 2.5. Mediation Analysis

Immune cells and inflammatory cytokines identified as having significant causal effects on ME/CFS in the two-sample MR analysis were further examined for causal relationships between them. If a significant causal relationship was detected, mediation analysis was conducted to evaluate whether inflammatory cytokines mediated the pathway from immune cells to ME/CFS.

## 3. Results

**Figure 2:**
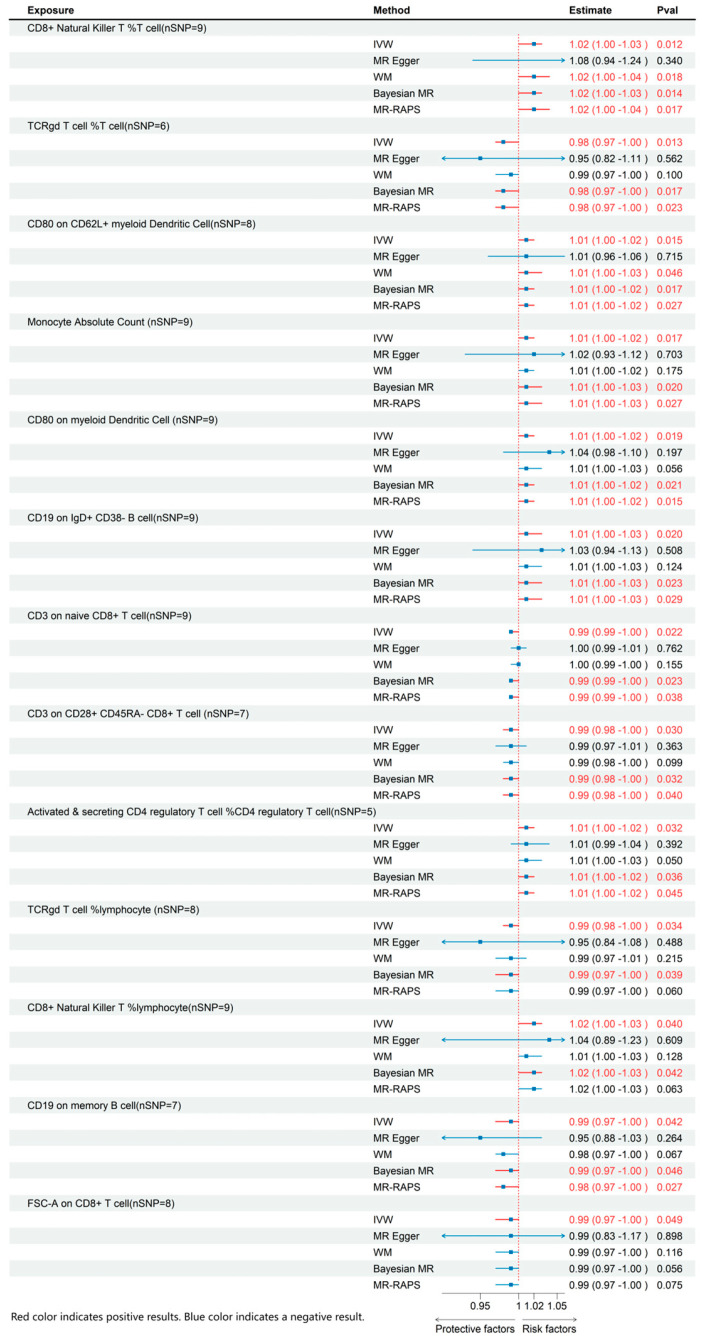
MR effects of immune cell on ME/CFS. The red color of the numbers and the red dotted line indicate a positive result.

**Figure 3:**
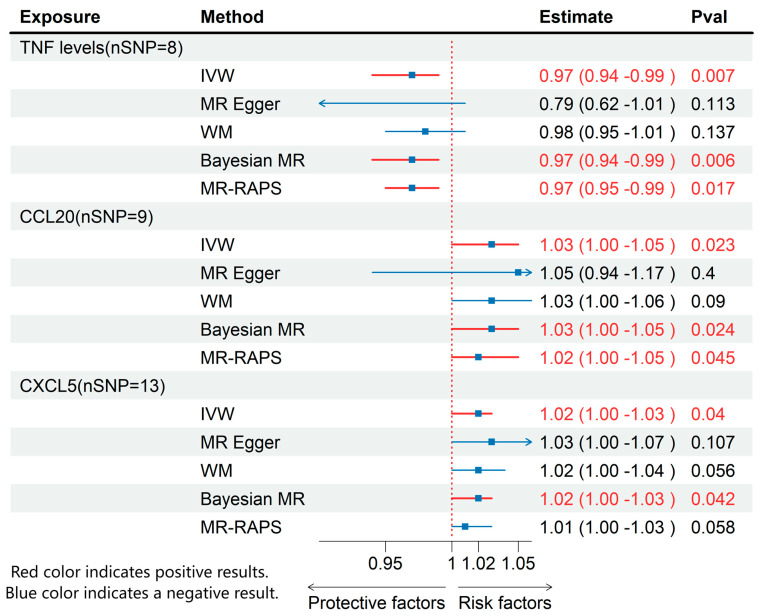
MR effects of circulating inflammatory cytokines on ME/CFS. The red color of the numbers and the red dotted line indicate a positive result.

Through MR analysis of circulating inflammatory cytokines and immune cell phenotypes, we identified 3 inflammatory cytokines and 13 immune cells that were causally associated with ME/CFS. Details of all the IVs used are given in Supplementary Tables S1–S3. Detailed results are presented in Figure 2 and Figure 3, with sensitivity analysis findings provided in Supplementary Table S4.

### 3.1. Effects of Immune Cell on ME/CFS

Regarding immune cell phenotypes (Figure 2), elevated levels of CD8+ natural killer T cells as a percentage of T cells and lymphocytes, CD19 on IgD+ CD38- B cells, absolute monocyte count, CD80 on CD62L+ myeloid dendritic cells, activated and secreting CD4 regulatory T cells as a percentage of CD4 regulatory T cells, and CD80 on myeloid dendritic cells were significantly linked to an increased risk of ME/CFS. Conversely, higher levels of CD3 on naive CD8+ T cell, CD3 on CD28+ CD45RA- CD8+ T cell, FSC-A on CD8+ T cell, TCRγδ T cell %lymphocyte, CD19 on memory B cell, and TCRγδ T cell %T cell were significantly linked to a decreased risk of ME/CFS.

### 3.2. Effects of Inflammatory Cytokines on ME/CFS

Using IVW method, we found that higher levels of TNF were associated with a decreased risk of ME/CFS (OR: 0.97; 95% CI: 0.94–0.99; *p* = 0.007), while increased levels of CXCL5 (OR: 1.02; 95% CI: 1.00–1.03; *p* = 0.04) and CCL20 (OR: 1.03; 95% CI: 1.00–1.05; *p* = 0.02) were associated with an elevated risk of ME/CFS (Figure 3). None of these cytokines exhibited pleiotropy or heterogeneity.

### 3.3. MR Results of Effects of Immune Cell on Inflammatory Cytokines

**Figure 4:**
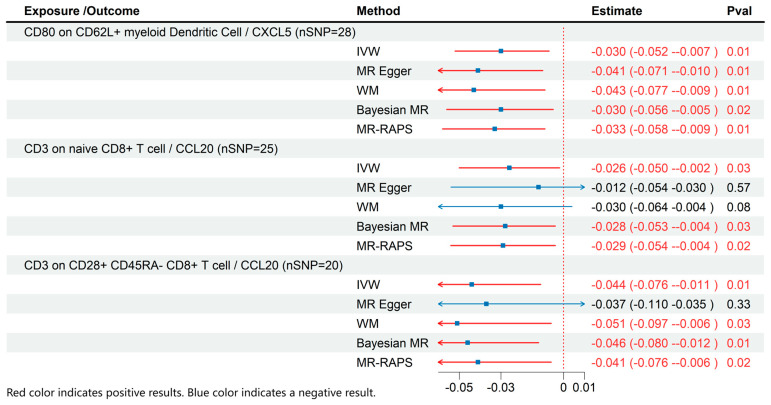
MR effects of immune cells on Inflammatory Cytokines. The red color of the numbers and the red dotted line indicate a positive result.

Based on the results of the previous analyses, we conducted additional MR analyses to evaluate immune cells and inflammatory cytokines with significant causal relationships to ME/CFS (Figure 4). The results demonstrated that CD80 on CD62L+ myeloid dendritic cells (β: −0.03; 95% CI: −0.052–−0.007; *p* = 0.01) was associated with reduced CXCL5 levels. Similarly, CD3 on naive CD8+ T cells (β: −0.026; 95% CI: −0.05–−0.002; *p* = 0.03) and CD3 on CD28+ CD45RA- CD8+ T cells (β: −0.044; 95% CI: −0.076–−0.011; *p* = 0.01) were associated with lower CCL20 levels.

### 3.4. Mediating Role of Circulating Inflammatory Cytokines

Expanding on the conclusions drawn from earlier analyses, we performed mediation analyses to explore possible intermediate effects, as shown in Table 2. Nevertheless, none of these mediation effects reached statistical significance, with all *p*-values exceeding 0.05. These findings imply that inflammatory cytokines are unlikely to play a significant role in mediating the connection between immune cells and ME/CFS. Although no significant mediating effects were found, it is possible that there are more complex indirect pathways that we have not yet detected, or that the mediating effects are only apparent at specific stages of disease progression, or that there may be some dose dependency, etc., which need to be elucidated by more in-depth studies.

## 4. Discussion

This is the inaugural MR examination, establishing a causal link among immune cells, inflammatory cytokines, and ME/CFS. Our study highlights CD8+ natural killer T cells as a percentage of T cells and lymphocytes, CD19 on IgD+ CD38- B cells, absolute monocyte count, CD80 on CD62L+ myeloid dendritic cells, activated and secreting CD4 regulatory T cells as a percentage of CD4 regulatory T cells, CD80 on myeloid dendritic cells, CXCL5, and CCL20 as key contributors to ME/CFS risk. Conversely, higher levels of CD3 on naive CD8+ T cell, CD3 on CD28+ CD45RA- CD8+ T cell, FSC-A on CD8+ T cell, TCRγδ T cell %lymphocyte, CD19 on memory B cell, TCRγδ T cell %T cell, and the inflammatory cytokine TNF were significantly linked to a decreased risk of ME/CFS. These results underscore the critical role of immune dysfunction in ME/CFS pathogenesis and provide essential insights into potential diagnostic biomarkers. This study uses MR analysis to offer strong evidence of the immune system’s role in ME/CFS, facilitating future research on targeted therapies.

The complicated pathophysiology of ME/CFS, a chronic, diverse illness, is still not fully understood. According to recent studies, ME/CFS is frequently associated with immune system abnormalities. Patients often exhibit significant T cell regulatory impairments, B cell proliferation, an abnormal increase in regulatory T (Treg) cell proportions, and markedly reduced cytotoxic activity of NK cells [28,29,30]. Additionally, it is believed that infections brought on by pathogens like Epstein–Barr virus (EBV) are a major factor in the development of ME/CFS [31]. γδ T cells (TCRγδ) represent a specialized subset of T cells that detect antigens through unique receptors and function by activating B cells or targeting EBV-infected cells for destruction [32,33,34]. Studies have found that defects in EBV-specific B memory cells and T memory cells in patients with ME/CFS may contribute to diminished viral clearance, suggesting that the activation of immune cells could be a critical aspect of ME/CFS treatment [35]. Recently, viral infection and ME/CFS studies have garnered attention. A systematic review and meta-analysis examined 64 trials with 18 viruses, including 4971 ME/CFS patients and 9221 controls. Two DNA viruses (human herpesvirus-7 and parvovirus B19) and three RNA viruses (Borna disease virus, enterovirus, and Coxsackievirus B) were substantially more prevalent in ME/CFS patients than in healthy and diseased controls [36]. The Ross River virus (RRV), an Australian zoonotic arbovirus, may also cause ME/CFS. RRV affects the host immune response through immune escape strategies, which may account for chronic idiopathic fatigue. These findings illuminate immune pathology and exhaustion after viral infections, particularly in cases of long COVID following SARS-CoV-2 infection [37].

Functional abnormalities in monocytes, T cells, NK cells, and myeloid dendritic cells are particularly pronounced in patients with ME/CFS. Single-cell RNA sequencing has revealed that monocyte dysregulation within patients correlates positively with disease severity [7]. The proportions and activities of T cell subsets, such as CD3+ and CD8+ T cells, are significantly reduced, while the proportion of Treg cells is abnormally elevated [38,39]. These changes may exacerbate the inflammatory state of ME/CFS by suppressing NK cell function and impairing antigen-presenting cell activity [30,39]. Treg cells notably suppress antigen-presenting cell function by interacting with CD80, CD86, and MHCII molecules on dendritic cells (DCs), which may lead to DC maturation defects and impact their antigen presentation and cytokine secretion by helper T (Th) cells [40,41]. The expression levels of genes linked to inflammation are closely linked to abnormal B cell proliferation, indicating that B cells may contribute to the pathogenic processes of ME/CFS by producing primary antigens [42]. Increased differentiation or defective activation of NK cells in response to external stimuli may affect NK cytotoxic activity through different mechanisms [43,44].

The presence of an inflammatory state in patients with ME/CFS is also noteworthy. ME/CFS is associated with increased pro-inflammatory cytokines, such as IL-6, TNF-α, and IL-1β, contributing to the chronic inflammatory state and disease pathogenesis [45,46]. While TNF-α levels are elevated in ME/CFS [47,48], our MR analysis suggests a protective genetic predisposition, potentially due to reverse causality or confounding factors in observational studies. MR isolates lifelong genetic effects, which may reflect distinct etiological pathways. TNF-α has been linked to cognitive dysfunction and musculoskeletal pain in ME/CFS patients [49,50]. Elevated NF-κB levels contribute to oxidative stress and chronic inflammation [51,52], with TNF-α playing a central role in these processes. Studies show a correlation between TNF-α and fatigue, particularly in leukemia and depression patients [53,54].

CXCL5, a chemokine involved in neutrophil recruitment, shows altered levels in systemic lupus erythematosus [55], but its role in ME/CFS requires further study. Similarly, CCL20, a significant chemotactic factor, is elevated in rheumatoid arthritis [56,57] and may have a similar role in ME/CFS. Elevated chemokines like CXCL5 and CCL20 are also observed in fibromyalgia, suggesting common immune activation mechanisms with ME/CFS [58]. Understanding the inflammatory mechanisms in ME/CFS may provide insights into its etiology and therapeutic targets [48,59].

In this study, a two-sample MR method with IVW was used for the analysis, and various methods such as the MR-Egger regression, WM, Bayesian weighted MR method, and MR-RAPS were also combined to ensure the robustness of the results. This approach of using multiple methods together allows for a better understanding of cause-and-effect relationships and helps address issues like pleiotropy and weak instrumental variables, making the results more trustworthy than in earlier studies. Also, while looking at how circulating inflammatory cytokines affect the link between immune cell profiles and ME/CFS, this study used mediation analysis to dig deeper into the biological processes involved, unlike many earlier studies that only focused on basic correlation or single-factor causal links without thoroughly investigating mediating effects.

This study, while robust, has certain limitations. MR analysis depends on genetic variants as instrumental variables, which may not be available for all immune cell phenotypes, potentially diminishing statistical power. Secondly, while efforts were made to adhere to MR assumptions, the possibility of genetic variants directly influencing ME/CFS or related diseases cannot be completely excluded, which may introduce bias. Addressing these limitations will require future studies to incorporate diverse analytical methods and expand population diversity to validate and generalize the findings. While MR minimizes confounding, modest effect sizes (e.g., CXCL5 OR = 1.02) may reflect polygenic contributions or residual pleiotropy. Replication in larger cohorts is critical. Finally, the ME/CFS data are derived from self-reports and are subject to potential classification errors, but the dataset is the current ME/CFS dataset with a large sample size.

## 5. Conclusions

To sum up, this study offers the first proof of a link between ME/CFS, inflammatory cytokines, and immune cells. Immune cell function dysregulation has been found to be a substantial risk factor for ME/CFS, highlighting its potential utility as a biomarker for the condition. Moreover, these findings nominate CXCL5/CCL20 as potential diagnostic biomarkers and TNF modulation as a therapeutic strategy. Clinical trials targeting B-cell activity (e.g., rituximab) or myeloid dendritic cell CD80 pathways warrant exploration. Future studies should aim to uncover the precise mechanisms underlying immune cell dysfunction and inflammatory processes, thereby facilitating the creation of personalized therapeutic approaches.

## Figures and Tables

**Table 1:** Datasets employed for MR analyses.

Phenotype	Data Source	Sample Size	PubMed ID	GWAS ID	Ancestry
Immune cell	Orrù V et al. [14]	3757	32929287	GCST90001391-GCST90002121	European
Inflammatory cytokines	Zhao JH et al. [15]	14,824	37563310	GCST90274758-GCST90274848	European
ME/CFS	UKB	462,933	NA	ukb-b-8961	European

NA stands for absence.

**Table 2:** MR results of mediation analysis.

Exposure	Mediation	Outcome	Mediated Effect	Mediated Proportion	*p*
CD3 on naive CD8+ T cell	CCL20	ME/CFS	−0.000659 (−0.00152, 0.000197)	10.2% (23.3%, −3.04%)	0.13
CD3 on CD28+ CD45RA- CD8+ T cell	CCL20	ME/CFS	−0.00111 (−0.00275, 0.00053)	12.3% (30.6%, −5.88%)	0.18
CD80 on CD62L+ myeloid Dendritic Cell	CXCL5	ME/CFS	−0.000442 (−0.00118, 3 × 10^−4^)	−3.48% (−9.31%, 2.35%)	0.24

## Data Availability

Data are contained within the article or Supplementary Materials.

## Supplementary Materials

The following supporting information can be downloaded at https://www.mdpi.com/article/10.3390/biomedicines13051200/s1: Table S1: Detailed information of instrumental variables used for immune cells on ME/CFS; Table S2: Detailed information of instrumental variables used for inflammatory cytokines on ME/CFS; Table S3: Detailed information of instrumental variables used for immune cells on inflammatory cytokines in mediation analysis; Table S4: Sensitivity analysis in the MR analysis.

## Abbreviations

The following abbreviations are used in this manuscript:

MR	Mendelian randomization
GWAS	Genome-wide association study
IV	Instrumental variable
SNP	Single nucleotide polymorphisms
IVW	Inverse variance weighted
WM	Weighted median
BWMR	Bayesian weighted MR
(ME/CFS)	Myalgic encephalomyelitis/chronic fatigue syndrome
OR	Odds ratios
CI	Confidence interval
